# In vivo*/*ex vivo efficacy of artemether–lumefantrine and artesunate–amodiaquine as first-line treatment for uncomplicated falciparum malaria in children: an open label randomized controlled trial in Burkina Faso

**DOI:** 10.1186/s12936-019-3089-z

**Published:** 2020-01-06

**Authors:** Moussa Lingani, Léa Nadège Bonkian, Isidore Yerbanga, Adama Kazienga, Innocent Valéa, Hermann Sorgho, Jean Bosco Ouédraogo, Petronella Francisca Mens, Henk D. F. H. Schallig, Raffaella Ravinetto, Umberto d’Alessandro, Halidou Tinto

**Affiliations:** 1 Institut de Recherche en Sciences de la Santé/Direction Régionale du Centre Ouest (IRSS/DRCO), Nanoro, Burkina Faso; 2 Unité de Recherche Clinique de Nanoro (URCN), Nanoro, Burkina Faso; 3 Unité de Recherche sur le Paludisme et Maladies Tropicales Négligées, Centre Muraz, Bobo-Dioulasso, Burkina Faso; 4 École de Santé Publique, Université Libre de Bruxelles, CP594, Route de Lennik 808, 1070 Bruxelles, Belgique; 5 Department of Medical Microbiology, Experimental Parasitology Unit, Amsterdam University Medical Centres, Academic Medical Centre at the University of Amsterdam, Amsterdam, The Netherlands; 6 Medical Research Council Unit, The Gambia, Disease Control & Elimination Theme, Fajara, The Gambia; 7 Public Health Department, Institute of Tropical Medicine, Antwerp, Belgium

**Keywords:** Artemisinin-based combination therapy, In vivo/ex vivo, Efficacy, Safety, Uncomplicated malaria, Paediatric, Sub-Saharan Africa, Burkina Faso

## Abstract

**Background:**

Artemisinin-based combination therapy (ACT) is recommended to improve malaria treatment efficacy and limit drug-resistant parasites selection in malaria endemic areas. 5 years after they were adopted, the efficacy and safety of artemether–lumefantrine (AL) and artesunate–amodiaquine (ASAQ), the first-line treatments for uncomplicated malaria were assessed in Burkina Faso.

**Methods:**

In total, 440 children with uncomplicated *Plasmodium falciparum* malaria were randomized to receive either AL or ASAQ for 3 days and were followed up weekly for 42 days. Blood samples were collected to investigate the ex vivo susceptibility of *P. falciparum* isolates to lumefantrine, dihydroartemisinin (the active metabolite of artemisinin derivatives) and monodesethylamodiaquine (the active metabolite of amodiaquine). The modified isotopic micro test technique was used to determine the 50% inhibitory concentration (IC50) values. Primary endpoints were the risks of treatment failure at days 42.

**Results:**

Out of the 440 patients enrolled, 420 (95.5%) completed the 42 days follow up. The results showed a significantly higher PCR unadjusted cure rate in ASAQ arm (71.0%) than that in the AL arm (49.8%) on day 42, and this trend was similar after correction by PCR, with ASAQ performing better (98.1%) than AL (91.1%). Overall adverse events incidence was low and not significantly different between the two treatment arms. Ex vivo results showed that 6.4% *P. falciparum* isolates were resistant to monodesthylamodiaquine. The coupled in vivo*/*ex vivo analysis showed increased IC50 values for lumefantrine and monodesethylamodiaquine at day of recurrent parasitaemia compared to baseline values while for artesunate, IC50 values remained stable at baseline and after treatment failure (p > 0.05).

**Conclusion:**

These findings provide substantial evidence that AL and ASAQ are highly efficacious for the treatment of uncomplicated malaria in children in Burkina Faso. However, the result of *P. falciparum* susceptibility to the partner drugs advocates the need to regularly replicate such surveillance studies. This would be particularly indicated when amodiaquine is associated in seasonal malaria chemoprophylaxis (SMC) mass drug administration in children under 5 years in Burkina Faso.

*Trial registration* clinicaltrials, NCT00808951. Registered 05 December 2008,https://clinicaltrials.gov/ct2/show/NCT00808951?cond=NCT00808951&rank=1

## Background

Although the global incidence of malaria has decreased with 41% and its related mortality with 62% between 2000 and 2015, *Plasmodium falciparum* malaria remains a deadly endemic parasitic disease with over 219 million new cases and above 435,000 deaths reported in 2017 [1]. Approximately 92% of the falciparum malaria cases occur in sub-Saharan Africa and 70% of them are children under 5 years of age [1]. The World Health Organization (WHO) guidelines recommend a 3-day regimen of artemisinin-based combination therapy (ACT) for the treatment of uncomplicated malaria, and ACT has become crucial in efforts to eliminate malaria [2]. The extensive deployment of ACT combined with vector control measures led to a substantial decrease in malaria transmission and its public health consequences [3]. However, these efforts could be jeopardized by the occurrence and spread of artemisinin resistance in *P. falciparum* malaria, as this was already reported in South-East Asia [4–8]. As of 2019, artemisinin resistance has been confirmed in six countries of the Greater Mekong Sub-region [9].

By analogy to the history of chloroquine resistance and recent reports of declining ACT efficacy in Africa, it can be predicted that ACT resistance may spread from South-East Asia to India and progressively to sub-Saharan Africa within few years [9, 10]. Indeed, declining parasitological response rates to treatment and sub-microscopic persistence of *P. falciparum* after treatment with ACT have been reported in Africa; possibly due to the emergence of parasites with reduced drug sensitivity [10–12]. Resistance to amodiaquine, the partner drug in the artesunate–amodiaquine (ASAQ) combination, mediated principally by mutations in 2 putative drug transporter genes, encoded by *Plasmodium falciparum chloroquine resistance transporter (pfcrt)* and *Plasmodium falciparum multidrug resistance protein 1* (*pfmdr1),* could be a possible cause [13], and such mutations are selected in infections following recent treatment with ASAQ [14, 15]. The resistance could spread given that amodiaquine is associated with sulfadoxine-pyrimethamine (AQ/SP) for the seasonal malaria chemoprophylaxis (SMC) adopted in many Sahelian countries, including Burkina Faso [16, 17]. Decreased susceptibility of *P. falciparum* isolates to lumefantrine was reported in African children particularly in those who were recently treated with artemether–lumefantrine (AL) [14]. This situation is of concern as ACT are currently the most potent drug available to treat uncomplicated malaria [18].

In Burkina Faso, standard treatment for uncomplicated malaria changed in 2005 from chloroquine to AL and ASAQ as first line treatments and since then, ACT coverage has improved [19]. While this improved coverage is welcome, it also bears the potential of resistant parasite selection as drug pressure, secondary to poor treatment practices, inadequate patient adherence to prescribed drug regimens, and usage of sub-standard forms of the drug, is a key factor in the emergence of resistance [20].

Given the history of resistance to the previous anti-malarial drugs, the presence of artemisinin-resistant *P. falciparum* malaria in South-East Asia and the risk that resistance appearing elsewhere reach sub-Saharan Africa, there is a continuous need for local and global surveillance to collect up-to-date information that will detect early signs of resistance and trigger national, regional and global action [21]. Therefore, the regular surveillance of *P. falciparum* sensitivity against artemisinin-based combinations in sub-Saharan Africa is needed. So far, apart from the standard in vivo drug efficacy recommended by the WHO for the assessment of anti-malarial drug [22], there are no validated molecular markers of resistance to artemisinin derivatives, although *P. falciparum* chromosome 13 (‘kelch’ motif or K13) (*pfk13*) was associated with slow in vivo parasite clearance in south east Asia [7–9]. In such context, the combined ex vivo and in vivo tests present a tool to regularly assessed ACT efficacy [23–26]. The combination of these tests offers a comprehensive assessment of the efficacy of anti-malarial dugs and enables the prediction of the possible emergence of drug resistance. This can subsequently aid the guidance of locally-adapted anti-malarial drug prescription policies. The purpose of this study was to assess the coupled ex vivo*/*in vivo efficacy of AL and ASAQ for the treatment of uncomplicated *P. falciparum* malaria in children from a semi-urban area of Burkina Faso.

## Methods

### Study design and sites

This was a phase IV, open label controlled trial, randomized two-parallels arms, using a modified WHO 2009 protocol for surveillance of anti-malarial drug efficacy [22]. The study was carried out in a semi urban area of Bobo-Dioulasso in western Burkina Faso between December 2008 and December 2010, 5 years after the country had adopted and implemented the new first-line treatment protocols for uncomplicated malaria which is still currently in use in the country. The study area is characterized by a climate of Sudano-Guinean type, with an alternating rainy season from June to November and dry season from December to May. Malaria is hyperendemic with seasonal transmission but can be perennial around rivers and rice paddies with peak transmission during the rainy season [27]. The commonest vectors are *Anopheles gambiae* sensu stricto (*s.s.*), *Anopheles funestus* and *Anopheles arabiensis*, and *P. falciparum* is the predominant malaria parasite [27–29].

### Patients and inclusion criteria

During the study period, all male and female patients aged 6 months to 15 years inclusive, with fever (axillary temperature ≥ 37.5 °C) or history of fever in the last 24 h, visiting the peripheral health centres of the Dafra health district catchment area were screened using light microscopy. Children weighing 5 kg or more, with a confirmed *P. falciparum* (parasitaemia ≥ 4000/μL to 200,000/μL) mono-infection, haemoglobin level above 5.0 g/dl, and agreed to participate whenever applicable by giving their assent and if their parents or guardian provided written informed consent. Patients were not included if they were not willing to participate or had participated to any drug trial within the last 30 days, or had known hypersensitivity to the study drug, or were severely malnourished (defined as weight for height < 70% of the median NCHS/WHO reference), or had severe malaria. Patients were excluded after randomization if slide re-evaluation demonstrated a parasite density or species outside the inclusion criteria, if the patient experienced repeated vomiting of study medications on day 0, anti-malarial drug intake outside the study protocol during the follow-up period, or a voluntary consent withdrawal. For each included participant, five millilitres of venous blood were collected in EDTA coated tubes (Turumo, Escap, Belgium) for ex vivo assessments.

### Laboratory procedures for parasitaemia, haemoglobin and polymerase chain reaction (PCR)

Blood smear were obtained to check the presence of *P. falciparum* and to estimate the parasitaemia at day 0 (before inclusion) and at each scheduled or unscheduled visit. Thick and thin blood films were prepared, dried and Giemsa-stained according to standard operating procedures and examined under light microscopy at 1000-time magnification. Parasite density was calculated by counting the number of asexual parasites per 200 white blood cells (WBC) in the thick blood film, based on an assumed WBC of 8000/μl. One hundred high-powered fields (HPF) were examined (independent of presence or absence of asexual parasite stages). The parasite density per microlitre was calculated using the following formula: W${\text{Parasite density}}/\upmu {\text{I}}\, = \,({\text{number of parasites counted}}\, \times \, 8000)/\left( {\text{number of leukocytes counted}} \right)$ Blood smears were examined by two readers and, in the case of discordant results, by a third reader. Discordant results were defined as a difference between the two readers in (a) *Plasmodium* species, (b) positive and negative, (c) with parasite > 400/μL; if the higher count divided by the lower count was > 2, (d) with parasite ≤ 400/μL; if the higher reading is > log10 higher than the lower reading. Haemoglobin level was measured at day 0, 14, 28, 42 and any unscheduled visit with a portable spectrophotometer (HemoCue, Ängelholm, Sweden).

From included patients, four drops of blood were collected on filter paper (Whatman grade 3), labelled, air-dried and stored in seal plastic bags at ambient temperature. Parasite DNA was subsequently extracted using the Qiagen spin column kit for dry blood spots. For patient that experience treatment failure after day 6, parasite collected at day 0 and at day of treatment failure were genotype in stepwise fashion using GLURP (glutamate rich protein), MSP1 (merozoite surface protein 1) and MSP2 (merozoite surface protein 2) according to the standard WHO protocols [30]. If for any of the 3 loci, an allele was not share between day 0 and time of treatment failure, the infection was classified as new infection. If at least one matched allele was found at every locus, the outcome was classified as a recrudescence.

### Ex vivo assays

Ex vivo tests were described elsewhere [31]. Briefly, isolates of *P. falciparum* (parasitaemia ≥ 4000/μL to 200,000/μL) were collected before treatment and at day of recurrent parasitaemia. Drug sensitivity tests were performed within 24 h of bleeding, without culture adaptation using the modified isotopic micro-test technique [25]. The drugs tested were lumefantrine (Novartis Pharma, Basel, Switzerland), dihydroartemisinin (Sigma Tau., Rome, Italy) and monodesethylamodiaquine (WHO/TDR, Geneva, Switzerland).

### Interventions and follow up

At inclusion (day 0), patients who met the eligibility criteria were randomly assigned to receive either AL (a fixed-dose combination of 20 mg artemether/120 mg lumefantrine; Coartem^®^ Novartis) or ASAQ (a fixed-dose combination of artesunate and amodiaquine at three different dosages; Coarsucam™, Sanofi Aventis). Treatment were allocated on a computer-generated randomization list basis provided by an off-site investigator. Opaque sealed envelopes were prepared according to the randomization list with unique codes corresponding to the allocated treatment. Treatment allocation was done only after all eligibility criteria were confirmed. According to weight-based guidelines, a study nurse supervised the oral administration of AL [1 (patient weight, 5–14 kg), 2 (15–24 kg), or 3 (25–34 kg) and 4 (≥ 35 kg) tablets twice daily for 3 days] with fatty food or ASAQ [1 tablet of 25 mg artesunate/67.5 mg amodiaquine (5–9 kg), 50 mg/135 mg (9–18 kg), or 100 mg/270 mg (18–36 kg) once daily for 3 days] with water.

In this study all the participants treatments were directly observed by a study nurse at the clinic in both groups. In the AL group where patients had two administration each day, patients who lived far from the clinic stayed for the evening dose. And those living close to the centre and who wished, were allowed to leave and return at the clinic for the evening dose. After study drug administration, all children were observed at the clinic for an hour. A full dose was repeated if vomiting occurred within 30 min of administration and halved if vomiting occurred between 30 min and 1-h. Children (or caregivers) were provided with paracetamol for treatment of febrile symptoms. Those with a haemoglobin level of < 10 g/dL were treated with ferrous sulphate and anthelmintic.

Patients were asked to return on days 1, 2, 3, 7, 14, 21, 28, 35, and 42, as well as any other day if they felt ill, for a standardized medical history and physical examination. Blood sampling by finger prick was carried out for thick blood smears and for storage on filter paper at day 7, 14, 21, 28, 35, 42 and any unscheduled day if they feel unwell. Haemoglobin level was measured on day 0, day 14, day 28, day 42 and at the time of unscheduled visits. Patients who did not return for follow-up were visited at home. Treatment failures were treated with quinine 10 mg/kg orally three times a day for 7 days. Patients who developed evidence of severe malaria (including those who’s haemoglobin dropped at a level < 5 g/dL) or danger signs (convulsions, lethargy, inability to drink or breast feed, repeated vomiting, inability to stand/sit due to weakness) were referred for treatment with parenteral quinine and supportive care at the Bobo-Dioulasso University Hospital. Patients were excluded from the per-protocol population in case they used other anti-malarial drugs outside of the study, serious adverse events requiring a change of treatment, withdrawal of informed consent, or loss to follow-up. At the end of 42-day follow up, treatment outcomes were assessed based on the WHO 2009 protocol for surveillance of anti-malarial drug efficacy: adequate clinical and parasitological response (ACPR), early treatment failure (ETF), late parasitological failure (LPF), and late clinical failure (LCF) (Additional file 1). Total treatment failure was calculated as the sum of ETF, LPF and LCF [22]. Adverse events and serious adverse events were recorded, assessed and follow-up throughout the trial and up to completion of each event. Adverse event was defined as any untoward medical occurrence in a patient administered a pharmaceutical product and which does not necessarily have a causal relationship with the treatment. A serious adverse event was any adverse event that resulted in patient death, was life-threatening, required inpatient hospitalization or a prolongation of an existing hospitalization, required an intervention to prevent permanent impairment or has caused congenital anomaly.

### Ethical considerations

Artemether–lumefantrine and artesunate plus amodiaquine are the first line anti-malarial treatments in Burkina Faso, so this was considered as a phase IV study with low-risk for participants. The study protocol was reviewed and approved by the institutional ethics committee of Centre Muraz in Burkina Faso (Deliberation 18-2009/CE-CM). Informed consent was obtained from individual study participants in the presence of an independent witness whenever the participant was illiterate. Children aged 12 years and over were asked to provide their assent if they were able to do so. Written informed consent was obtained from the guardians for all children before entering the study. The study was conducted according to the WHO Good Clinical Practices guidelines and according to Burkina Faso national regulations.

### Statistical analysis

AL and ASAQ were adopted by Burkina Faso National Malaria Control Programme in 2005 on a non-inferiority basis with both treatments used as alternative first-line treatment for uncomplicated malaria. In the current phase IV study, it was hypothesized that the efficacy of each study treatments is likely to be at least 90%. Therefore, 220 children per arm would be able to show at the 5% significance level with 95% power, and a maximum loss to follow up of 10%, that the difference in efficacy between treatments is not more than 10%.

Data were double entered and verified with Microsoft Access 2007 and analysed with Stata 15 software (StataCorp. TX, USA). Efficacy and safety data were evaluated using a modified intention-to-treat analysis that included all enrolled patients and also using a per-protocol analysis included all participants who did not experience any major protocol violation. Risks of new infections and recrudescence during the 42-day follow-up were estimated using the Kaplan–Meir product-limit formula and comparisons of cumulative risk with the non-parametric log-rank test. Data were censored for patients who did not complete the follow-up and for infections due to another *Plasmodium* species. Other categorical variables were compared with Chi square or Fisher exact test, and continuous variables were compared using the independent-samples t*-*test. The ex vivo data management and statistical methods are described elsewhere [31]. A *p* value of < 0.05 was considered statistically significant.

## Results

### Trial subjects

**Fig. 1:**
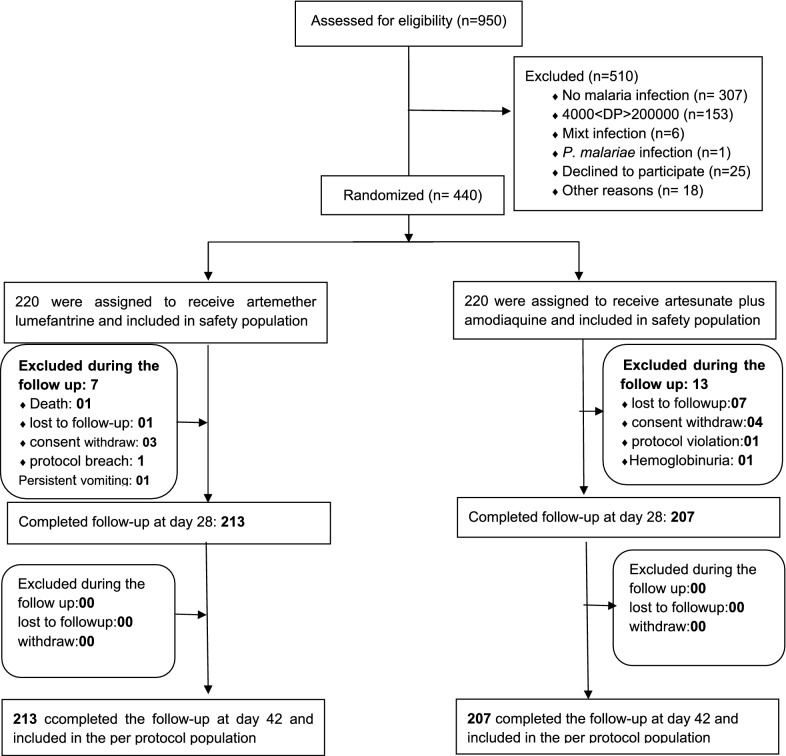
Trial profile: 42-day follow-up of study participants by treatment arm at the Dafra health district medical centre, Burkina Faso 2008-10. *AL* artemether–lumefantrine, *ASAQ* artesunate–amodiaquine

Figure 1 summarizes the trial profile. Out of 950 children aged between 6 months and 15 years screened for a suspicion of malaria, 643 (67.7%) were positive for falciparum malaria. In total, 440 children fulfilling the eligibility criteria were recruited and randomized to receive either AL or ASAQ at a ratio of 1:1. At the end of the 42 days follow up period, 213 in the AL arm and 207 in the ASAQ arm completed the study. One patient with a negative blood smear on third and 7th day of follow-up died before the day 14 follow up visit for a suspicion of meningitis. Eight participants were lost to follow-up, and other seven participants stopped their participation as their caregivers moved outside the study catchment area. Four participants were excluded from the per-protocol population: one participant for persistent vomiting, two participants for taking anti-malarial drugs outside the study protocol, and the other one for severe malaria with haemoglobinuria. Baseline characteristics including mean age, height, and mean geometric parasite density were similar for children assigned to the 2 study arms excepted for the gender ratio with however no significant impact on outcome analysis (Table 1).

**Table 1:** Baseline characteristics of the study participants by treatment arm at the Dafra health district medical centre, Burkina Faso, 2008–2010

Intention to treat population mean ± standard deviation	ASAQ N = 220	AL N = 220	Total N = 440
Gender m/f, %	45.4/54.6	55.6/44.6	50.5/49.6
Age, year	6.4 ± 3.2	6.4 ± 3.1	6.5 ± 3.1
Height, cm	114.3 ± 19.4	114 ± 19.5	114.15 ± 19.4
Weight, kg	19.1 ± 7.5	18.9 ± 8.0	19.01 ± 7.7
GMPD, parasitaemia/µl	44567	47074	45803

*AL* artemether–lumefantrine, *ASAQ* artesunate plus amodiaquine, *GMPD* geometric mean of parasite density

### In vivo efficacy of trial regimens

The large majority of treatment failures were late parasitological failures, with fewer children having a late clinical failure (Table 2). In per protocol population (N = 420 participants 213 in the AL arm and 207 in the ASAQ arm), the PCR-unadjusted cure rate at day 42 was significantly higher in the ASAQ arm (71.0%) than in the AL arm (49.8%) [risk difference = − 22.0; 95% CI − 31.1; − 12.9 (p < 0.001)]. Trend remained similar, after adjustment by PCR, between ASAQ (98.1%) and AL (91.1%) [risk difference = − 7.0; 95% CI − 11.0; − 2.7 (p < 0.001)].

**Table 2:** PCR-adjusted and unadjusted cure rates of the study participants at day-42 at Dafra health district medical centre, Burkina Faso, 2008–2010

Outcome: day 42	AL	ASAQ	Difference (CI 95%)	p-value
Variables				
PCR corrected efficacy outcome—no. (%)				
*Per protocol* population (N = 420) n (%)	*213*	*207*	**–**	**–**
Adequate clinical and parasitological response	194 (91.1)	203 (98.1)	− 7 (− 11.0 to − 2.7)	< 0.0006
Early treatment failure	0 (0.0)	0 (0)	–	–
Late clinical failure	0 (0.0)	0 (0)	–	–
Late parasitological failure	19 (8.9)	4 (1.9)	–	–
Total number of failure	19 (8.9)	4 (1.9)	–	–
Not corrected for reinfection n (%)				
Adequate clinical and parasitological response	106 (49.8)	147 (71.0)	− 22 (− 31.1 to − 12.9)	< 0.0001
Early treatment failure	0 (0)	0 (0)	–	–
Late clinical failure	32 (15.0)	22 (10.6)	–	–
Late parasitological failure	75 (35.2)	38 (18.4)	–	–
Total number of failure	107 (50.2)	60 (28.9)	–	–
Intent-to-treat population (N = 440)	*220*	*220*	**–**	**–**
PCR-adjusted				
Total number of patients n	194	203		
Adequate clinical and parasitological response % (95% CI)	88.2 (83.2–91.8	92.3 (87.9–95.2)	− 4.1 (− 9.8 to − 1.5)	0.148
PCR-unadjusted				
Total number of patients n	106	147	–	–
Adequate clinical and parasitological response % (95% CI)	48.2 (41.6–54.8)	66.8 (60.3–72.8)	− 18.6 (− 27.4 to 9.4)	< 0.001

*AL* artemether–lumefantrine, *ASAQ* artesunate–amodiaquine, CI 95% confident interval, *n* number, % percentage

In intent-to-treat population (N = 440 participants 220 in the AL arm and 220 in the ASAQ arm), the PCR-unadjusted cure rate at day 42 was still significantly higher in the ASAQ arm (66.8%) than in the AL arm (48.2%) [risk difference = − 18.6; 95% CI − 27.4; − 9.4 (p < 0.001)]. However, difference of PCR adjusted cure rate was not statistically significant between ASAQ (92.3%) and AL (88.2%) [risk difference = − 4.1; 95% CI − 9.8; − 1.5 (p = 0.148)].

**Fig. 2:**
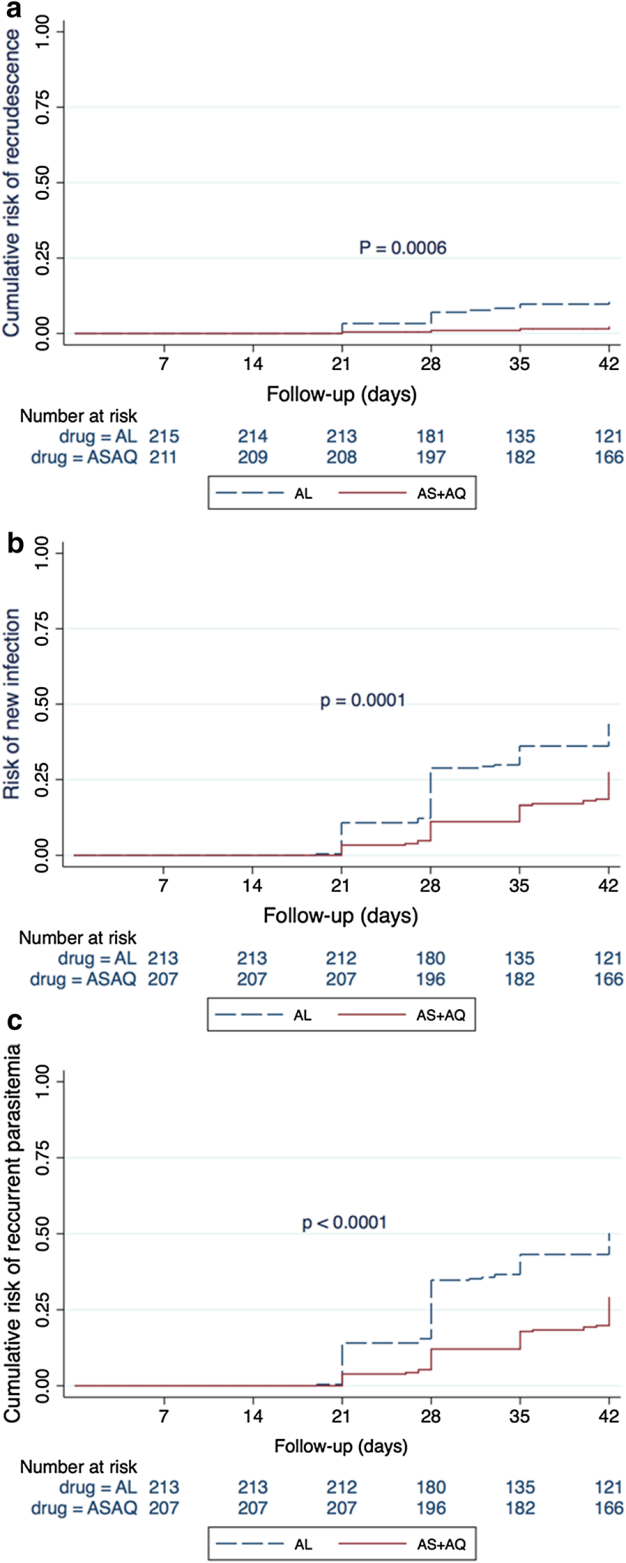
Kaplan Meier curves showing the treatment failure cumulative proportion for each treatment arm by day 42 in the per protocol population (N = 420): **a** recrudescent infections, **b** new infection, **c** recurrent infections (Recrudescent plus new infection)

New infection started to appear after day 14, first in the AL arm and then in ASAQ arm and (the difference) was statistically significant at day 42 (p < 0.001) (Fig. 2a). However, recrudescence started to appear after day 21, first in the AL arm and then in the ASAQ arm and this (difference) was statistically significant at day 42 (p < 0.001) (Fig. 2b). The rate of treatment failure (new infection and recrudescent was significantly lower in the ASAQ arm than in the AL arm (p < 0.001) (Fig. 2c). After the completion of treatment (day 3), almost all the participants (418/420, 99.5%) had negative blood smear (two patients in the AL group had positive blood smears).

At day 0, approximately 6.8% (15/220) in AL arm and 4.5% (10/220) in ASAQ arm had positive blood smear for gametocyte (p > 0.05). Gametocytes clearance was slower in ASAQ arm (8% positive) than in AL arm (2.8%) at day 7 (p = 0.03), and total clearance was observed at day 42. The fever clearance was rapid in both treatment arms, and no patient was febrile on day 3 following the treatment initiation. The average increase in haemoglobin level at day 42 was significantly higher compared to baseline in the two arms and there was no statistically significant difference between both arms (0.9 g/dL in ASAQ versus 0.7 g/dL in the AL arm; P > 0.05).

Both treatments were well-tolerated with no significant difference in the incidence of adverse events (AE) (Table 3). However, one fatal case of suspected cerebrospinal meningitis in AL arm and one case of severe malaria with haemoglobinuria in ASAQ arm were recorded as serious adverse events.

**Table 3:** Safety and tolerability outcome

Adverse events	AS + AQ n (%)	AL n (%)	p-value
Overall	88 (40.0)	87 (39.5)	1.00
Specific AEs			
Abdominal pain	18 (8.1)	17 (7.7)	1.00
Digestive (nausea, vomiting, anorexia, diarrhea)	22 (10.0)	22 (10.0)	0.86
Cough and rhinitis	23 (10.5)	28 (12.7)	0.55
Bronchitis	12 (5.4)	13 (5.9)	1.00
Fever	40 (18.2)	47 (21.4)	0.47
Headache	16 (7.3)	21 (9.5)	0.49
Other events	17 (7.7)	12 (5.5)	0.44
SAE of any cause	1 (0.5)	1 (0.5)	1.00

*AE* adverse event, *SAE* serious adverse event, *AL* artemether lumefantrine, *ASAQ* artesunate–amodiaquine

### Ex vivo efficacy of study drug active metabolites

Table 4 summarizes the mean 50% inhibitory concentration (IC50) of monodesethylamodiaquine (MDA), the active metabolite of amodiaquine, dihydroartemisinin (DHA), the active metabolite of artemisinin derivatives and lumefantrine (Lum) at day 0 as baseline values, and the proportion of resistant isolates, i.e. IC50 above the cut-off of resistance. Cut-off value was set at 60 nM for MDA. However, there was no cut-off values for DHA and lumefantrine and, therefore, the resistance rates could not be determined for these drugs. The overall resistance rate to MDA was 6.4%.

**Table 4:** Mean values of 50% inhibitory concentration of anti-malarial drug at day 0

Anti-malarial	IC50 Geometric mean (nmol/l) [IC 95%]	Range		Resistant isolates n (%)
		Min	Max	
Monodesethylamodiaquine	19.30 (18.04–20.65)	0.81	595.93	24 (6.37)
Dihydroartemisinin^a^	0.83 (0.76–0.89)	0.15	38.73	–
Lumefantrine^a^	25.12 (22.40–28.16)	0.77	166.1	–

^**a**^No cut-off value for resistance defines

### Coupled in vivo*/*ex vivo efficacy AL and ASAQ in children

**Fig. 3:**
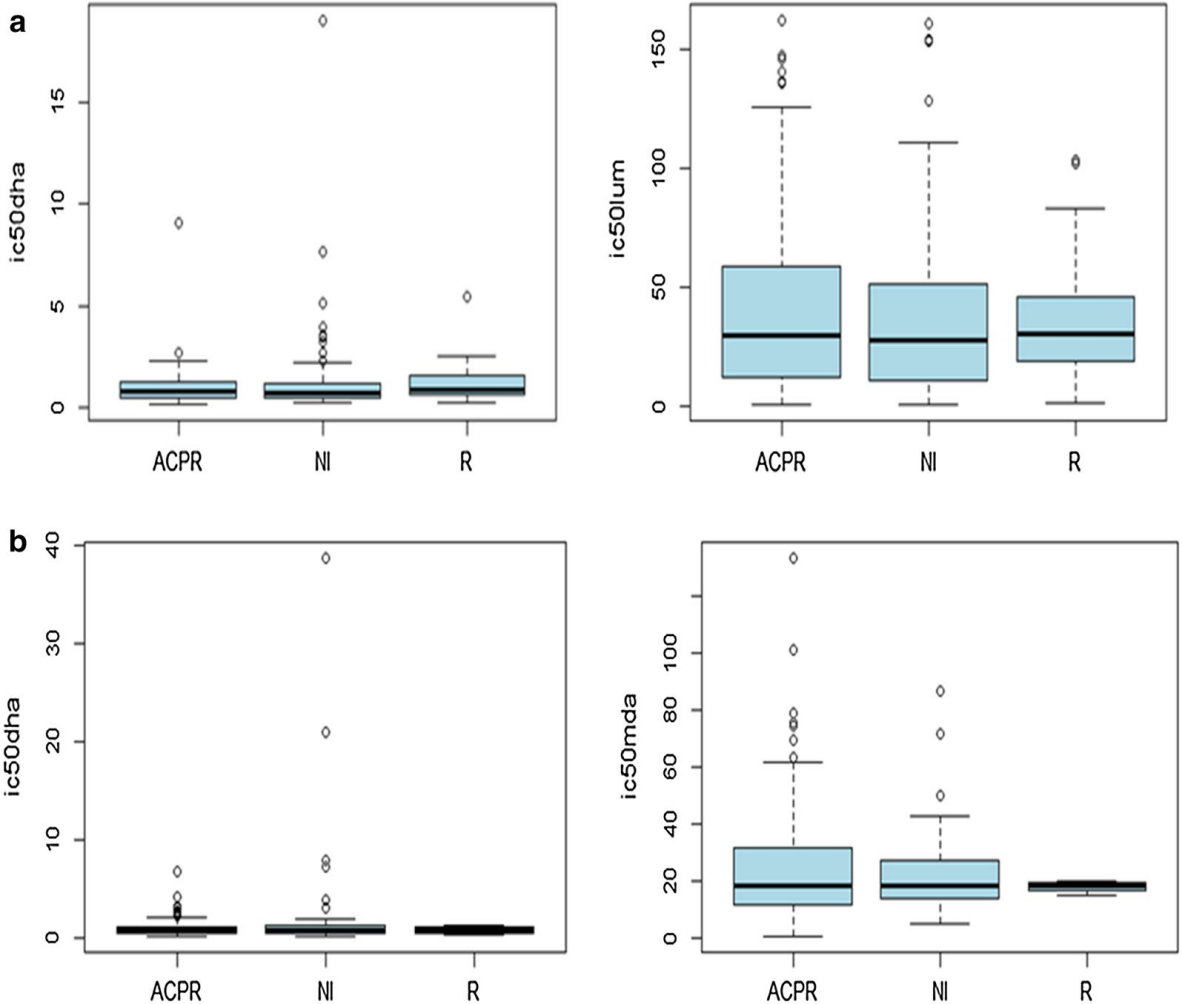
Mean geometric IC50 values at day 0 and treatment outcome in study participants at the Dafra health district medical centre, Burkina Faso 2008–2010: **a** artemether–lumefantrine arm, **b** artesunate amodiaquine arm. *D0* day of inclusion before treatment administration, *NI* new infection, *R* recrudescent, *ACPR* adequate clinical and parasitological response, *DHA* dihydroartemisinin, *Lum* lumefantrine, *MDA* monodesethylamodiaquine, *IC50* 50% inhibitory concentration

From the 24 isolates (6.4%) resistant to MDA at inclusion, the treatment outcome resulted in one recrudescent case, 11 ACPR cases, and 12 new infection cases. Figure 3 compares the mean IC50 values at inclusion between participants with adequate clinical and parasitological response (ACPR) and patients with treatment failure. The IC50 values at inclusion could not adequately predict the treatment outcome during the follow-up period as mean values were similar between the ACPR, the recrudescence and the new infection group.

In the ASAQ arm, mean IC50 values were not significantly different on day of treatment failure compare to baseline (p > 0.05) for MDA and DHA (Table 5). Similarly, in the AL arm, the mean IC50 values were not significantly different between day of treatment failure and baseline for DHA, although for lumefantrine the increase was particularly high (p = 0.05) (Table 5).

**Table 5:** Mean geometric IC50 values for each component of AL and ASAQ at D0 (before treatment) versus day of treatment failure among treatment participants

Treatment	n	n	P value
	IC50 at D0 in nM (IC 95%)	IC50 at DoR in nM (IC 95%)	
ASAQ			
MDA	188	33	–
	19.80 (17.83–21.99)	24.94 (20.68–30.08)	0.94
DHA	190	34	–
	0.85 (0.76–0.95)	0.66 (0.47–0.93)	0.96
AL			
DHA	191	54	–
	0.8 (0.72–0.90)	0.67 (0.54–0.83)	0.43
LUM	192	53	–
	24.13 (20.45–28.46)	37.07 (28.37–48.44)	0.05

nM nanomolar, *DoR* day of recurrent parasitaemia, *ASAQ* amodiaqine–artesunate, *AL* artemether lumefantrine, *MDA* monodesethylamodiaquine, *DHA* dihydroartermisinin, *LUM* lumefantrine

## Discussion

In this study, the efficacy and safety of the two artemisinin-based combinations used as first-line treatment for uncomplicated malaria in Burkina Faso were investigated. Noteworthy their importance for malaria management, amodiaquine the partner drug in the ASAQ combination is used in the seasonal malaria chemoprevention strategy although there is a WHO recommendation discouraging such use of first-line drug component in mass drug administration within the same zones [32]. Given that no efficacy study assessing the two combinations was conducted during the last 10 years in the country; these results, even if dating back to 2010, can still bring useful information to policymakers as it combines both ex vivo and in vivo data.

Both AL and ASAQ were highly effective in clearing malaria infections with PCR adjusted cure rates over 90%, a threshold required by the WHO for an anti-malarial to be considered effective [33]. However, ASAQ presented a higher cure rate than AL although the difference was within the pre-specified 10% points margin of equivalence. Therefore, based on these data, the choice of the two artemisinin-based combinations as first-line treatment for uncomplicated *P. falciparum* malaria was appropriate in Burkina Faso. Efficacy of both treatment was evaluated in other sub-Saharan African countries and similar good efficacy were reported in west Africa [34–38], and in eastern Africa [11, 39]. In the current study, both treatments were equally efficacious with however, most treatment failure observed in AL arm (8.9%). The possible declining in AL efficacy should alert the national malaria program to undertake more frequent surveillance. In particular, the data presented here refer to the years 2010, so such a phase IV study would be now timely in Burkina Faso. Noteworthy, a previous study conducted in the same area, when the new protocol was not yet scaled-up, reported better cure rates with AL (95.9%) and ASAQ (96.1%) [40]. Within 5 year, an important decline was reported in the efficacy rate of AL from 95.9% to 91%. Similar trend was observed in Liberia, a west African country, where high efficacy rates were reported for ASAQ (99.1%) and AL (94.2%) before the policy change [41]. By contrast, in Eastern Africa AL was more efficacious than ASAQ even though the differences were not statistically significant. Indeed, Mårtensson et al. in their study in Tanzania reported higher efficacy of AL (94%) than AS + AQ (91%) [42]. In the Democratic Republic of Congo, Epsié et al. reported higher efficacy of AL (99.1%) than AS + AQ (98.3%) [43]. This contrasting results between Western Africa and Eastern Africa could be explained by the better efficacy of amodiaquine the partner drug in the ASAQ combination in Western Africa than Eastern Africa, and could be the reason behind the better efficacy of artesunate-amodiaquine in this study [44, 45]. Both regions have also high *pfcrt* 76T prevalence, with however a rapid decline in Eastern Africa than in Western Africa and this varying prevalence of *pfcrt* 76 T could influence the efficacy of the combination [46].

In the same period as in this study, ASAQ (89.7%) and AL (89.8%) presented lower efficacy rate in Nanoro, northern part of the country with a different malaria transmission pattern [47]. Although in the present study all treatments administrations were directly observed, this alone cannot explain this difference as the cure rate in this study remain higher in the intent-to-treat population. The difference in the malaria transmission profiles (Sudano-Sahelian type of climate with shorter transmission season than in the western part) between the two regions could be behind the observed difference as geographic variations in ACT efficacy were already reported elsewhere [48].

From a clinical point of view, any recurrent parasitaemia should be treated with an efficacious anti-malarial regardless whether it is recrudescence or new malaria infection, so the most practical means of assessing these drugs may be to evaluate their impact on recurrent malaria. By this measure, recurrent parasitaemia rates were high in both arms with however a remarkably higher rate in the AL arm (50.2%). Most studies in sub-Saharan Africa reported similar results with up to 40% of recurrent parasitaemia [35, 40, 42, 49]. The duration of post treatment prophylaxis is however an important criterium in the choice of anti-malarial drugs, especially in areas with a high risk of re-infection. These post treatment prophylactic effects are mainly related to the elimination half-life of the partner drug. Lumefantrine has the shortest elimination half-life (3–5 days) [50], then followed by monodesethylamodiaquine (10–18 days) [51], and could be the reason behind the better efficacy of artesunate plus amodiaquine. However, a prolonged elimination and protection time may drive the development of resistance in a setting of high malaria transmission due to frequent and longer exposition of parasite to decreased drug concentration level in patient blood [52].

The main goal of the coupled in vivo*/*ex vivo analysis was to predict the treatment outcome based on the values of the IC50 at baseline. However, this was not possible within this study as only one (1/24) patient resistant to monodesethylamodiquine at day 0 was a true treatment failure. This raises the issue of defining appropriate IC50 thresholds to adequately predict the in vivo outcomes.

The mean IC50 values was higher at recurrent parasitaemia than at day 0 for both artemether lumefantrine and artesunate amodiaquine. If that increase was not statistically significant for monodesethylamodiaquine, due possibly to the limited number of recrudescence in the artesunate–amodiaquine arm, it was however high for lumefantrine. A prior study in east Africa reported a significant directional selection for *Pfmdr1* 184F in recurrent malaria infections after treatment with AL in Uganda [48]. This evidence highlight the dynamic evolutionary status of the susceptibility of *P. falciparum* to lumefantrine, particularly in patients recently treated with AL. This advocate for a regular surveillance of lumefantrine in the AL combination. Although both AL and ASAQ performed well with few recrudescence, changes in the relative performance of the 2 partner drugs should draw the attention of policymakers, especially considering the very high incidence of malaria in Burkina Faso. Remarkably, nearly half of children treated with AL experienced recurrent malaria within 42 days. This risk was 40% lower in children treated with ASAQ, suggesting important benefits of this regimen. However, these data and prior results suggest that widespread use of ASAQ may rapidly select parasites with reduced susceptibility [14–16]. This is quite alarming as amodiaquine is used for the seasonal malaria chemoprophylaxis in sub-Sahel countries [53, 54].

Both drugs presented a good safety profile with low incidence of adverse events. Fever and headache were common in both treatment arm and were likely to be symptoms of the malaria infection. Cough was also frequent in both arm and may be related to additional bacterial infections as clinical bronchitis was also a common finding. Abdominal pain, anorexia and diarrhoea were relatively common in both arms and were related to the malaria infection. Two cases of serious adverse events were reported in this study.

The first case was about a 5 years of age female child, weighing 18 kilograms of body mass, high fever (39.5 ℃) with a baseline parasitaemia of 127,788/µl, an inclusion haemoglobin level of 9.8 g/dL and who received his first dose of ASAQ, presented 12 h later a febrile status associated to a diuresis of 250 ml of brownish urines. The diagnosis of haemoglobinuria was suspected by study physician and participant was discontinued from the study treatment and referred to Bobo-Dioulasso university hospital where the diagnosis was confirmed. Participant was managed at the hospital with intravenous artesunate and recovered before day 7. This occurrence was classified as related to the study drug by the study physician.

The second case was a 5 years old male child, weighing 12 kilograms, with high inclusion fever (40.5 ℃), a haemoglobin level of 7.9 g/dL, an inclusion parasite level of 104, 301 asexual parasites/µl, treated with AL and well recovered with negative blood smear on day of study completion and confirmed on day-7 follow-up. The patient missed his day-14 scheduled visit and an investigator visited his caregivers home and was informed that patient presented a febrile neurological syndrome on day-12 after inclusion, managed with herbal therapy without improvement and was referred to another health centre for a febrile loss of consciousness. The diagnostic of bacterial meningitis was suspected and treated using intravenous ceftriaxone, blood transfusion and supportive care. The meningitis diagnostic could not be confirmed due unsuccessful lumbar puncture and subsequent child death. The serious adverse event was classified as not related to the study treatment.

## Conclusion

Both artemether–lumefantrine and artesunate amodiaquine are appropriate as first-line treatment of uncomplicated malaria in Burkina Faso, based on the findings of this phase IV study since both presented PCR adjusted cure rates above 90%. However, the post-treatment prophylaxis was unsatisfactory for both treatment particularly for artemether–lumefantrine and advocate regular surveillance of the efficacy of the two combinations. Given that these data are a bit old, it seems timely to conduct new surveillance study in the country to produce updated information. In addition, any alternative drug should be investigated.

## Supplementary information

**Additional file 1.** The document contains the definitions of study outcomes according to the WHO criteria (WHO 2003).

## Data Availability

The dataset used and analysed during the current study is available from the corresponding author.

## Abbreviations

ACT: artemisinin-based combination therapy
ASAQ: artemether–lumefantrine
AL: artesunate–amodiaquine
AQ/SP: amodiaquine–sulfadoxine–pyrimethamine
DHA: dihydroartemisinin
MDA: monodesethylamodiaquine
Lum: lumefantrine
CI: confidence interval
PCR: polymerase chain reaction
CRUN: clinical research unit of Nanoro
SMC: seasonal malaria chemoprophylaxis
WHO: World Health Organization
*pfcrt*: *Plasmodium falciparum chloroquine resistance transporter*
*pfmdr1*: *Plasmodium falciparum* multidrug resistance protein 1
ACPR: adequate clinical and parasitological response
ETF: early treatment failure
LPF: late parasitological failure
LCF: late clinical failure
WBC: white blood cells
HPF: high-powered fields
GLURP: glutamate rich protein
MSP1: merozoite surface protein 1 gene
MSP2: merozoite surface protein 2 gene
AE: adverse event
SAE: serious adverse event
IC50: 50% inhibitory concentration
nM: Nanomolar
DoR: day of recurrent parasitaemia
DNA: de-oxy-ribonucleic-acid
GMPD: geometric mean parasite density
