# Supplementary material for: In vivo/ex vivo efficacy of artemether–lumefantrine and artesunate–amodiaquine as first-line treatment for uncomplicated falciparum malaria in children: an open label randomized controlled trial in Burkina Faso
Source: Malar J. 2020 Jan 6;19:8. doi: 10.1186/s12936-019-3089-z (PMC6945612; doi:10.1186/s12936-019-3089-z)
Supplement: Supplementary file 1 — Additional file 1. The document contains the definitions of study outcomes according to the WHO criteria (WHO 2003). [file 12936_2019_3089_MOESM1_ESM.docx]

**Appendix 1: Definition of study outcomes**

Treatment failure (TF) is defined according to the WHO criteria (WHO 2003) as the sum of early* and late** treatment failures.

* Early Treatment Failure (ETF) (one of the following)

(i) Development of danger signs or severe malaria (see Appendix V) on Day 0, Day 1, Day 2 or Day 3, in the presence of parasitaemia,

(ii) Parasite density on Day 2 > Day 0 count, irrespective of axillary temperature,

(iii) Presence of parasitaemia on Day 3 with fever (axillary temperature ≥ 37.5°C),

(iv) Parasitaemia on Day 3 ≥ 25 % of count on Day 0.

** Late treatment failure (LTF)

LTF is divided in late clinical and late parasitological failure.

Late Clinical Failure (LCF):

(i) Development of danger signs or severe malaria after Day 3 in the presence of parasitaemia, (See Appendix V for the criteria of severe malaria/danger signs).

(ii) Presence of parasitaemia and fever on any day after Day 3, without having previously meet the criteria of ETF.

Late Parasitological Failure (LPF):

Reappearance of parasitaemia after day 3 in the absence of fever (axillary temperature <37.5°C) without having previously meet the criteria of ETF or LCF.

The adequate clinical and parasitological response (ACPR) is 1-TF (unadjusted or adjusted). It is defined as absence of parasitaemia at the end of the follow up period (day 42), irrespective of axillary temperature without previously meeting any of the criteria of early and late treatment failure. In the adjusted estimates, patients with late asexual parasite reappearance (with or without fever) will be considered ACPR if the PCR analysis shows a
